# No association between*P53*codon 72 polymorphism and risk of squamous cell carcinoma of the head and neck

**DOI:** 10.1054/bjoc.1999.0993

**Published:** 2000-02

**Authors:** N Hamel, M J Black, P Ghadirian, W D Foulkes

**Affiliations:** Montreal General Hospital Research Institute,Department of Head & Neck Surgery and Cancer Prevention Research Unit, Sir MB Davis – Jewish General Hospital, McGill University, Montreal, Quebec, H3T 1E2, Canada; Epidemiology Research Unit, Research Centre, Hotel Dieu Campus, CHUM, Montreal, Quebec, Canada

**Keywords:** *P53*, polymorphism, head and cancer, squamous cell, HPV, low penetrance

## Abstract

An initial report suggested that patients homozygous for the arginine allele at codon 72 of*P53*were at increased risk for human papillomavirus (HPV)-related cervical cancer, but other groups have not confirmed this finding. Since approximately 18–36% of head and neck cancers are HPV-related, we examined the genotypic frequencies at that locus in 163 cases with squamous cell carcinoma of the head and neck (SCCHN) and 163 ethnically matched controls. We found no significant excess of arginine homozygotes in cases compared to controls (*P*= 0.50). No significant differences in allele frequencies were observed when the data were stratified by tobacco exposure or by cancer site. These findings suggest a limited role, if any, for this*P53*polymorphism in SCCHN.© 2000 Cancer Research Campaign


British Journal of Cancer (2000) 82(4), 757–759
© 2000 Cancer Research Campaign
DOI: 10.1054/ bjoc.1999.0993, available online at http://www.idealibrary.com on IDEAL®

Short Communication

### No association between *P53* codon 72 polymorphism and risk of squamous cell carcinoma of the head and neck

**N Hamel[1], MJ Black[2], P Ghadirian[4] and WD Foulkes[1,3]**

[1]Montreal General Hospital Research Institute, [2]Department of Head & Neck Surgery and [3]Cancer Prevention Research Unit, Sir MB Davis – Jewish General Hospital, McGill University, Montreal, Quebec, Canada, H3T 1E2; [4]Epidemiology Research Unit, Research Centre, Hotel Dieu Campus, CHUM, Montreal, Quebec, Canada

**Summary** An initial report suggested that patients homozygous for the arginine allele at codon 72 of *P53* were at increased risk for human papillomavirus (HPV)-related cervical cancer, but other groups have not confirmed this finding. Since approximately 18–36% of head and neck cancers are HPV-related, we examined the genotypic frequencies at that locus in 163 cases with squamous cell carcinoma of the head and neck (SCCHN) and 163 ethnically matched controls. We found no significant excess of arginine homozygotes in cases compared to controls ($P$= 0.50). No significant differences in allele frequencies were observed when the data were stratified by tobacco exposure or by cancer site. These findings suggest a limited role, if any, for this *P53* polymorphism in SCCHN. © 2000 Cancer Research Campaign

**Keywords:** *P53*; polymorphism; head and cancer; squamous cell; HPV; low penetrance

Infection with human papillomavirus (HPV) has been implicated in more than 90% of cervical carcinomas in which specific viral oncogenes have been shown to integrate into the host genome and thereby deregulate cell proliferation. One such oncogene, *E6*, interferes with proper cell growth regulation by promoting the degradation of the tumour suppressor gene product P53 (zur Hausen, 1996). A recent report by Storey et al (1998) presented data suggesting that homozygosity for the allele encoding arginine at the polymorphic codon 72 of *P53* correlated with increased risk of HPV-associated cervical cancer. These results were obtained from genotyping 30 cervical tumours and 41 normal controls, with an observed Arg/Arg frequency of 76% in cancer patients compared to a frequency of 37% in controls. In addition, the data were supported by results from an in vitro assay demonstrating that the p53Arg form of the protein was more susceptible to degradation by the HPV E6 oncoprotein than the p53Pro variant.

HPV infection has also been correlated with oral cancer development. The overall prevalence of HPV in head and neck tumours is estimated to range between 18.5% and 35.9% (McKaig et al, 1998) depending on detection methods. The HPV types most frequently associated with head and neck cancer are the same as those primarily found in cervical cancer (i.e. HPV16 and HPV18). The existence of a similar risk factor for a given *P53* genotype in HPV-related oral cancer would facilitate screening of HPV-infected individuals by focusing screening efforts on high-risk individuals. We therefore examined the importance of this *P53* polymorphism with respect to the risk of cancer development in patients with squamous cell carcinoma of the head and neck (SCCHN).

*Received 22 March 1999*
*Revised 6 September 1999*
*Accepted 23 September 1999*

*Correspondence to:* WD Foulkes, Room L10-116, Montreal General Hospital, 1650 Cedar Avenue, Montreal, Quebec, Canada, H3G 1A4

### MATERIALS AND METHODS

Patients and controls examined in this study were interviewed between 1994 and 1998 and were matched on the basis of similar ethnic origin to control for population variations in allele frequencies. A questionnaire recording lifetime tobacco exposure was completed by all participants. DNA was extracted from blood using standard methods. Polymerase chain reaction (PCR) amplification was performed using standard buffer, nucleotides and enzyme supplied by Pharmacia Biotech (Upsala, Sweden) and the following primers: 4L1, 5′-GCAACTGACCGTGCAAGTCA-3′; 4U1, 5′-ATCTACAGTCCCCCTTGCCG-3′ (Sheldon Biotechnology Center, Montreal, Quebec, Canada). Cycling was performed as follows: 3 min denaturing at 95°C; 35 cycles of 30 s denaturation at 95°C, 30 s annealing at 60°C, 30 s extension at 72°C; 10 min incubation at 72°C. Restriction digestion with *Bst*U1 was performed using half the PCR reaction and according to the manufacturer's instructions (New England Biolabs Inc., Beverly, MA, USA). Digested samples were separated on 1.8% agarose-TBE (Tris–borate EDTA) gels, stained with ethidium bromide and photographed under UV. Statistical analysis was performed using Arcus QuickStat (Biomedical). We used either the $R \times C\ \chi^2$ test for association or Fisher's exact test to analyse our data. Gart's method was used to construct exact confidence limits for the odds ratio of the fourfold tables. With unmatched study design and a sample size of 163 cases, matched 1:1 to controls with $\alpha = 0.05$, we have an 80% power to detect an odds ratio (OR) of 2.0 or greater for the development of SCCHN in association with the Arg/Arg genotype. The OR observed by Storey et al (1998) was 5.7. All $P$-values are 2-sided.

### Results

We genotyped 163 incident and prevalent cases of SCCHN and 163 ethnically matched healthy controls. Cancer sites included the

**Table 1** R72P *P53* polymorphism genotype distribution in ethnically matched cases and controls

| | Arg/Arg (%) | Arg /Pro (%) | Pro/Pro (%) | Total (%) |
|---|---|---|---|---|
| Cases | 88 (54.0) | 68 (41.7) | 7 (4.3) | 163 (100) |
| Controls | 95 (58.3) | 61 (37.4) | 7 (4.3) | 163 (100) |

R × C $\chi^2$ = 0.64; *P* = 0.72. Fisher's exact test (Pro/Pro and Arg/Pro combined): *P* = 0.50 (OR = 0.84, 95% CI 0.75–1.89).

**Table 2** Genotypic frequency in control individuals from differing ethnic backgrounds

| Ethnicity/ Country of origin | Arg/Arg (%) | Arg/Pro (%) | Pro/Pro (%) | Total (%) |
|---|---|---|---|---|
| British | 24 (63.2) | 14 (36.8) | 0 (0.0) | 38 (100.0) |
| French Canadian | 37 (54.4) | 25 (36.8) | 6 (8.8) | 68 (100.0) |
| Jewish | 18 (60.0) | 12 (40.0) | 0 (0.0) | 30 (100.0) |
| Others | 16 (59.3) | 10 (37.0) | 1 (3.7) | 27 (100.0) |

R × C $\chi^2$ = 6.60; *P* = 0.36.

lip (*n* = 2), the tongue (*n* = 29), various mouth parts (*n* = 38), the pharynx (*n* = 24), the nasal cavity (*n* = 4) and the larynx (*n* = 66). The arginine form of codon 72 contains a *Bst*U1 site that is not present in the proline allele. We digested a 380-bp PCR fragment amplified from leuckocyte DNA with the enzyme *Bst*U1 and separated the fragments by agarose gel electrophoresis. The arginine allele, when cleaved by the *Bst*U1 enzyme, yielded two fragments of 210 and 170 bp, while the proline allele remained as a 380-bp band on the gel. No difference between genotypic frequencies in SCCHN cases and controls was observed (R × C $\chi^2$ = 0.64; *P* = 0.72) (Table 1). We then combined Pro/Pro homozygotes with Arg/Pro heterozygotes and the data was further analysed by Fisher's exact test to determine whether cases showed a significant excess of Arg/Arg homozygotes compared to controls, as was reported previously, but no significant difference was observed (*P* = 0.50).

The purpose of ethnically matching controls to cases is to prevent possible differences in allelic frequencies among various ethnic groups from resulting in biased genotypic distributions. In order to determine whether such ethnic differences exist for this polymorphism, we examined genotypic frequencies in our control group with respect to ethnic background using the R × C $\chi^2$ method. We found no significant difference in the genotypic distributions of the various ethnic groups ($\chi^2$ = 6.61; *P* = 0.36) (Table 2).

Differences in HPV positivity between cases and controls and by head and neck cancer site could hide a genotypic bias in HPV-infected patients, since about 18–36% of cases are expected to be positive for HPV, depending on the techniques used (McKaig et al, 1998). We do not have HPV typing information for cases or controls. Interestingly, both a positive (Schwartz et al, 1998) and an inverse (Portugal et al, 1997) correlation between heavy smoking (the most significant risk factor for SCCHN development) and the presence of HPV in host DNA has been reported. Schwartz et al (1998) also found that the oropharynx and the tonsils were the head and neck sites most likely to harbour HPV. This concurs with other data (McKaig et al, 1998). In light of these results, we analysed our data in two ways. We first divided our cases into four quartiles based on their pack year values. There was no significant difference in genotypic frequency between cases with these four different levels of tobacco exposure ($\chi^2$ = 6.91; *P* = 0.33) (Table 3). We also compared the genotypic frequencies of the groups with the lowest and highest exposure levels between which, based on previous results (Portugal et al, 1997; Schwartz et al, 1998), the greatest variation in the proportion of HPV-related cancers should be observed. We saw no significant difference in the frequency of arginine homozygotes in cases with the lowest and highest pack year values (*P* = 0.18, Table 3). There was slight evidence for a linear trend towards the proline allele being over-represented in cases with heavy tobacco exposure, but 4/12 cells have an expected *n* < 5. When we dichotomized the data at the median pack-year exposure for controls as well as cases, there was no difference in the OR between the lowest and highest exposed groups (1.19 and 1.09 respectively (non-significant), *P* = 0.85 for a test that the data are derived from different populations, data not shown). It should be noted that compared with the control group, the Arg/Arg allele frequency (the presumed disease-associated genotype) was not significantly elevated in any subgroup of SCCHN.

**Table 3** Effect of tobacco exposure level on genotypic distribution in cases

| Pack years | Arg/Arg (%) | Arg/Pro (%) | Pro/Pro (%) | Total (%) |
|---|---|---|---|---|
| 0.0–18.9 | 24 (58.5) | 17 (41.5) | 0 (0.0) | 41 (100.0) |
| 19.0–39.0 | 24 (58.5) | 16 (39.1) | 1 (2.4) | 41 (100.0) |
| 40.0–60.8 | 23 (57.5) | 14 (35.0) | 3 (7.5) | 40 (100.0) |
| 61.0–153.7 | 17 (41.5) | 21 (51.2) | 3 (7.3) | 41 (100.0) |

R × C $\chi^2$ = 6.91; *P* = 0.33. There is a linear trend of borderline significance (*P* = 0.057). Dichotomizing at the median pack-year exposure of cases diminished the significance of this result (*P* = 0.12). R × C $\chi^2$ (Pro/Pro and Arg/Pro combined to test for excess Arg/Arg) = 3.47; *P* = 0.32. Fisher's exact test comparing lowest and highest quartiles (Pro/Pro and Arg/Pro combined): *P* = 0.18 (OR = 1.99, 95% CI 0.76–5.27).

We then examined polymorphic frequencies by cancer sites. Although there are great discrepancies from one study to another, overall levels of HPV infection appear to be twice as high in cancers of the oral cavity as they are in the larynx (Franceschi et al, 1996; McKaig et al, 1998) (in contrast to the situation with laryngeal papillomatosis). Therefore we analysed the data without the larynx cancer cases (*n* = 97). The results were similar to those observed when the larynx cancer cases were included, and no differences in genotype frequencies were observed ($\chi^2$, *P* = 0.54, data not shown). When we analysed oropharynx and tonsillar cancers separately, no differences in genotype frequencies were observed (Arg/Arg = 8 and Arg/Pro = 6), with this small sample size (*n* = 14) we had very limited power to detect significant differences.

### DISCUSSION

We report here *P53* codon 72 polymorphism frequencies in SCCHN. We did not observe a significant difference between SCCHN cases and ethnically matched controls, nor did we find varying genotypic frequencies among any subgroup of cases. Following the earlier report (Storey et al, 1998), other studies of cervical cancer cases which ascertained larger groups of cases and controls have failed to uncover any significant overrepresentation of the Arg heterozygous genotype in controls (Helland et al, 1998;

 © 2000 Cancer Research Campaign

Hildesheim et al, 1998; Josefsson et al, 1998; Minaguchi et al, 1998). In addition, in a follow-up study originally designed to confirm the results presented by Storey et al (1998), genotyping of a larger group of controls and of additional cervical cancer cases (pooled with those cases previously reported) did not support the original observation, mainly due to a dramatic change in allelic frequencies in the control group. A genotypic distribution similar to ours was found in both cases and controls (Arg/Arg = 54%, Arg/Pro = 40%, Pro/Pro = 6% in cases; 63% Arg/Arg, 31% Arg/Pro, 6% Pro/Pro in controls) (Rosenthal et al, 1998). This is clear evidence that using controls derived from the same population as the cases and adequate sizing of the control group are of crucial importance to the validity of genotyping studies. Moreover, it is uncertain which is the best technique to use to detect clinically significant HPV infection of the head and neck (McKaig et al, 1998). Detection of HPV DNA in tumour tissue, which is the approach used in most studies to determine HPV infection status, may not be the best way to record on-going and persistent infection (Schwartz et al, 1998). The relationship between the codon 72 polymorphism in *P53* and HPV-positive SCCHN will not be finally determined until the genotype at codon 72 is correlated with the expression status of HPV in cases and controls, at each SCCHN site. Nevertheless, our preliminary results suggest it is unlikely that the Arg/Arg genotype of the *P53* codon 72 polymorphism constitutes an increased risk factor in HPV-associated SCCHN.

### ACKNOWLEDGEMENTS

We would like to thank Dr A Storey for providing us with controls. This work was funded in part by a Quebec Family Cancer Network grant from the Fonds de la Recherche en Santé du Québec (FRSQ) and by the Cancer Research Society Inc. WDF is a Chercheur Clinicien of the FRSQ.

### REFERENCES

Buchan I (1998) *Arcus QuickStat (biomedical) Version 1.2*. Addison Wesley Longman: Cambridge

Franceschi S, Munoz N, Bosc XF, Snijders PJ and Walboomers JM (1996) Human papillomavirus and cancer of the upper aerodigestive tract: a review of epidemiological and experimental evidence. *Cancer Epidemiol Biomarkers Prevent* **5**: 567–575

Helland Å, Langerød A, Hilde J, Olsen AO, Skovlund E and Børresen-Dale A-L (1998) P53 polymorphism and risk of cervical cancer. *Nature* **396**: 530–531

Hildesheim A, Schiffman M, Brinton LA, Fraumeni JF Jr, Herrero R, Bratti MC, Scharwtz P, Mortel R, Barnes W, Greenberg M, McGowan L, Scott DR, Martin M, Herrera JE and Carrington M (1998) P53 polymorphism and risk of cervical cancer. *Nature* **396**: 531–532

Josefsson AM, Magnusson PKE, Ylitalo N, Quarforth-Tubbin P, Pontén J, Adami HO and Gyllensten UB (1998) P53 polymorphism and risk of cervical cancer. *Nature* **396**: 531

McKaig RG, Baric RS and Olshan AF (1998) Human papillomavirus and head and neck cancer: epidemiology and molecular biology. *Head Neck* **20**: 250–265

Minaguchi T, Kanamori Y, Matsushima M, Yoshikawa H, Taketani Y and Nakamura Y (1998) No evidence of correlation between polymorphism at codon 72 of p53 and risk of cervical cancer in Japanese patients with human papillomavirus 16/18 infection. *Cancer Res* **58**: 4585–4586

Portugal LG, Goldengerg JD, Wenig BL, Ferrer KT, Nodzenski E, Sabnani JB, Javier C, Weichselbaum RR and Vokes EE (1997) Human papillomavirus expression and p53 gene mutation in squamous cell carcinoma. *Arch Otolaryngol Head Neck Surg* **123**: 1230–1234

Rosenthal AN, Ryan A, Al-Jehani RM, Storey A, Harwood CA and Jacobs IJ (1998) P53 codon 72 polymorphism and risk of cervical cancer in UK. *Lancet* **352**: 871–872

Schwartz SM, Daling JR, Doody DR, Wipf GC, Carter JJ, Madeleine MM, Mao EJ, Fitzgibbons ED, Huang S, Beckmann AM, McDougall JK and Galloway DA (1998) Oral cancer risk in relation to sexual history and evidence of human papillomavirus infection. *J Natl Cancer Inst* **90**: 1626–1636

Storey A, Thomas M, Kalita A, Harwood C, Gardiol D, Mantovani F, Breuer J, Leigh IM, Matlashewski G and Banks L (1998) Role of a p53 polymorphism in the development of human papilloma-virus-associated cancer. *Nature* **393**: 229–234

zur Hausen H (1996) Papillomavirus infections – a major cause of human cancers. *Biochem Biophys Acta* **1288**: F55–F78

*© 2000 Cancer Research Campaign*

